# Thermal versus cold instrument incisions in cervical dissection for oral squamous cell carcinoma: A comparative study

**DOI:** 10.6026/973206300212112

**Published:** 2025-07-31

**Authors:** Sunil Kumar Gulia, Ramanand O.V, Uday Sagar Sandepogu, Priya Sharma, Yadavalli Guruprasad, Palanati Sai Ram, Heena Dixit

**Affiliations:** 1 Department of Oral and maxillofacial Surgery, SGT University, Gurugram, Badli, Jhajjar, Haryana, India; 2 Department of Oral snd Maxillofacial Pathology, Nimra Institute of Dental Sciences, Ibrahimpatnam, Vijayawada, India; 3 Department of Oral and Maxillofacial Surgery, Consultant Maxillofacial Surgeon and Implantologist, St Theresa Hospital, Hyderabad India; 4 Department of Oral and Maxillofacial Surgery, Peoples dental Academy, People's University, Bhopal, Madhya Pradesh, India; 5 Department of Oral and Maxillofacial Surgery, Government Dental College and Research Institute, VIMS Campus, Cantonment, Ballari, India; 6 Department of Oral and Maxillofacial Surgery, MNR Dental College and Institute, Sangareddy, Telangana, India; 7 Hospital and Healthcare Management, DY Patil Deemed to be University, Navi Mumbai, India

**Keywords:** Oral squamous cell carcinoma, neck dissection, electrocautery, cold scalpel, wound healing

## Abstract

The impact of thermal versus cold instrument incisions in cervical dissection is of interest. A prospective comparative research was conducted on 40 patients with histopathologically confirmed OSCC undergoing neck dissection. EC significantly reduced incision time (7.4 ± 1.2 vs. 10.1 ± 1.6 min) and blood loss (142.5 ± 30.2 vs. 198.4 ± 35.7 mL). However, postoperative pain was higher in the EC group (VAS 6.8 ± 0.7 vs. 5.2 ± 0.9) and wound healing was delayed (11.2 ± 1.3 vs. 9.1 ± 1.4 days). No significant difference in operative duration or oncologic margin status was noted. While EC offers intraoperative advantages, CS incisions yield better postoperative healing and lower pain.

## Background:

Oral squamous cell carcinoma (OSCC) remains one of the most prevalent malignancies affecting the head and neck region, particularly in South Asia, where tobacco use, alcohol consumption and poor oral hygiene are prominent risk factors [1]. Surgical resection remains the cornerstone of curative treatment for OSCC, often involving cervical lymphadenectomy to manage regional metastasis and ensure clear margins [2]. The choice of surgical instruments during neck dissection has long been debated, particularly between conventional CS and modern thermal devices such as monopolar "electrocautery (EC)" or ultrasonic harmonic scalpels [3]. CS incisions are traditionally valued for their precision and minimal thermal damage, allowing for cleaner histopathological interpretation and potentially better wound healing outcomes [4]. However, thermal instruments have gained popularity for their ability to provide hemostasis, reduce intraoperative bleeding and shorten operative time [5]. Despite these advantages, concerns remain regarding increased lateral thermal injury, delayed wound healing and potential compromise in oncological safety margins associated with thermal devices [6]. Recent studies have suggested that thermal incision methods may cause increased tissue necrosis and impaired lymphatic drainage, potentially influencing postoperative morbidity, such as seroma formation or delayed healing [7]. Comparative research on the impact of incision technique on postoperative outcomes, including wound healing time, intraoperative blood loss, operative duration, pain scores and histological clarity, is still evolving [8]. While technological advances in surgical equipment promise efficiency, the priority in oncological surgery remains complete tumor clearance with minimal morbidity [9]. It is thus essential to investigate whether the benefits of thermal instruments outweigh their possible drawbacks in cervical dissection for OSCC. This research aims to comparatively evaluate cold steel versus thermal instrument incisions in neck dissection for OSCC, focusing on operative parameters, healing outcomes and pathological integrity of surgical margins [10]. Therefore, it is of interest to evaluate and clarify which technique optimally balances surgical efficiency with oncologic and patient-centered outcomes.

## Materials and Methods:

This prospective, comparative research was conducted in the Department of Oral and Maxillofacial Surgery at a tertiary care center over a period of 18 months. The research included 40 patients diagnosed with histo-pathologically confirmed OSCC scheduled for primary tumor resection along with cervical lymphadenectomy. Patients were randomly allocated into two equal groups (n=20 each) based on the type of surgical incision used for neck dissection: Group A underwent CS dissection, while Group B underwent thermal incision using monopolar EC. Inclusion criteria were patients aged 18-70 years with stage I-III OSCC, ECOG performance status ≤2 and no prior radiation or chemotherapy. Exclusion criteria included recurrent tumors, systemic infections, bleeding disorders, or immuno-compromised status. All surgeries were performed by the same surgical team to reduce operator variability. Intraoperative parameters such as incision time, total blood loss and operative duration were recorded. Postoperative parameters including pain scores (using VAS), wound healing status, drainage output and complications (*e.g.*, seroma, infection) were documented on days 1, 3, 7 and 14. Histopathological evaluation of margins and lateral thermal damage was conducted by a blinded pathologist. Data were analyzed using SPSS version 26. Statistical significance was set at p < 0.05.

## Results:

A total of 40 patients were enrolled, with 20 each in the CS (Group A) and EC (Group B) arms. The baseline characteristics, including age, gender, tumor site and TNM stage, were comparable between the two groups (p > 0.05), ensuring homogeneity for valid comparison. The mean incision time was significantly shorter in the EC group (7.4 ± 1.2 minutes) compared to the CS group (10.1 ± 1.6 minutes), with p = 0.001. EC also significantly reduced intraoperative blood loss (mean 142.5 ± 30.2 mL vs. 198.4 ± 35.7 mL, p = 0.002). However, the total operative time did not differ significantly between the two groups (p = 0.324) (Table 1 - see PDF). On postoperative day 1, pain scores were significantly higher in the EC group (VAS score 6.8 ± 0.7) than in the CS group (5.2 ± 0.9), p < 0.001. Wound healing, measured by days to epithelialization, was delayed in the EC group (mean 11.2 ± 1.3 days) compared to the CS group (9.1 ± 1.4 days), p = 0.004. Drain output on day 1 was also higher in the EC group. Complications such as seroma formation were more frequent in Group B (3 cases vs. 1 case), but not statistically significant (p = 0.291) (Table 2 - see PDF). These findings suggest that while EC offers faster incision and reduced bleeding, it may be associated with higher early postoperative pain and delayed wound healing compared to CS dissection.

## Discussion:

The comparative analysis of CS and EC techniques in cervical dissection for OSCC highlights key differences in intraoperative efficiency and postoperative healing. EC demonstrated a significantly shorter incision time and reduced intraoperative blood loss, supporting earlier evidence that thermal instruments provide superior hemostasis and procedural speed by simultaneously cutting and coagulating tissues [11]. These advantages can be particularly valuable in lengthy or complex oncologic surgeries, where minimizing blood loss is critical for maintaining hemodynamic stability and improving surgical visibility [12]. Despite these intraoperative benefits, EC use was associated with higher early postoperative pain scores and delayed epithelialization. These findings are consistent with prior studies that suggest thermal instruments generate lateral tissue damage and protein denaturation, which may provoke a more pronounced inflammatory response and delay the natural wound healing cascade [13]. Furthermore, greater drain output and a non-significant trend toward higher seroma formation in the EC group imply a potential compromise in lymphatic vessel integrity, which has also been noted in other head and neck surgeries utilizing thermal modalities [14]. From an oncological perspective, both techniques maintained clear margins with no significant difference in pathological outcomes, indicating that either method is oncologically safe when used with appropriate technique. However, histological assessment of margins may be more challenging when thermal damage distorts tissue architecture, a concern that emphasizes the need for surgical precision, especially in close-margin cases [15]. The clinical decision between CS and EC should thus be based on a balance of factors: while EC offers practical advantages during surgery, CS incisions appear to provide more favorable postoperative outcomes in terms of pain and healing. For patients with comorbidities or at high risk of wound healing complications, CS may be the preferred modality. In contrast, for cases requiring rapid control of bleeding or in resource-limited settings, EC may offer practical benefits. Overall, these findings reinforce the need for individualized surgical planning. Further studies with larger samples and long-term follow-up could provide additional insights into recurrence rates, functional recovery and scar quality, thus helping to refine surgical protocols for OSCC management [16, 17, 18, 19- 20].

## Conclusion:

We show that EC in cervical dissection for OSCC reduces incision time and blood loss but is associated with increased postoperative pain and delayed healing. CS incisions, though more time-consuming and associated with higher intraoperative bleeding, offer better outcomes in wound recovery and patient comfort. Both techniques are oncologically safe when margins are meticulously maintained.
